# Supplementary material for: Prevalence of the Frank’s sign by aetiopathogenic stroke subtype: A prospective analysis
Source: PLoS One. 2021 Dec 15;16(12):e0261080. doi: 10.1371/journal.pone.0261080 (PMC8673637; doi:10.1371/journal.pone.0261080)
Supplement: Attachment — Submitted filename: Comments.docx [file pone.0261080.s002.docx]

Comments to PONE-D-21-20627

ABSTRACT

- “Many studies have associated this sign with heart disease…” - I suggest you to be more precise in abstract and change it to “coronary artery disease”.

INTRODUCTION

- In the *Introduction* you stated: “The Frank’s sign or ear lobe crease (ELC) is a cutaneous clinical sign (…) covering at least 1/3 of the ear lobe”. I definitely do not agree with that sentence. There is no one clear definition and many authors utilized various ones, including many thresholds of relation of earlobe crease to the whole earlobe. I suggest you to mention this issue in your article.
- “Several studies have considered a possible link between ELC and CAD, and many studies have shown that ELC can serve as a marker of atherosclerotic disease and as a sign of an elevated risk of coronary heart disease in asymptomatic individuals [3-5].” – I suggest you to cite one of the recent reviews on this topic. References 3 to 5 are from 1977, 1974, and 1983, and many new articles have been published since then.
- In the *Introduction* you raised the topic of pathophysiology and development of ELC. I suggest you to read the new, interesting article (Stoyanov, et al. The Histological Basis of Frank's Sign. Head Neck Pathol. 2021;15(2):402-7. doi: 10.1007/s12105-020-01205-4) about it. You may consider to add it to references.
- “Some studies have described an association between Frank's sign and the presence of diabetes, hypertension, myocardial infarction and coronary artery disease in patients of both sexes.” – in reference number 4 there is “the prevalence of diabetes mellitus and hypertension was similar in both those with and without the ear crease” and in reference number 9: “Among the different CRFs significant associations were found only between ELC and hypertension (p<0.001) and diabetic retinopathy (p<0.05)”.
- “However, only a few studies have focused on the association between the presence of ELC and cerebrovascular disease.” – add references, please.
- “Additionally, carotid artery intima-media thickness (IMT) and atherosclerotic plaques in the carotid arteries have also been described in association with ELC.” – add reference, please.
- “Therefore, according to these findings, the presence of ELC could serve as a reliable marker of systemic atherosclerosis [11-15].” – reference number 13 (Ntaios, G. Embolic stroke of undeterminated source. Jacc. 2020;75:333-340) seems to be not related.

MATERIAL AND METHODS

- What was the time range when patients had been enrolled? Add this in *Material and methods* section, please.
- What were the “potential confounding factors for the evaluation of ELC…”. Specify this in *Material and methods* section, please.
- What do you exactly mean by “principal”, “secondary”, and “tertiary” variables in *Material and methods* section?
- I suggest you to add references to NIHSS, mRS.
- Is the protocol of this study available online?

STATISTICAL ANALYSIS

- “Results are expressed as (…) mean ± standard deviation (SD) for continuous variables depending on whether or not they are normally distributed.” – so, how did you presented continuous variables not normally distributed?
- In *Statistical analysis* section you noted: “The importance of ELC in all stroke subtypes and specifically in ESUS was assessed by binary logistic regression analysis…” and then in Table 1 in *Results* there is “Adjusted mean difference (95% IC)” that is other measure than ORs. Could you explain it to me?

RESULTS

- There is one crucial error in calculations presented through the whole manuscript. In *Abstract* you stated: “The Frank’s sign (…) was more prevalent in patients with an ischemic stroke (92.0%). Similar prevalence was found in the different ischaemic stroke subtypes.”. Then, in *Results* section you continued with: “There were no relevant differences in the prevalence of ELC by stroke subtypes.”, “In comparison with ischaemic stroke, we found a low prevalence of the Frank’s sign in haemorrhagic stroke (92.0% vs. 8.0%, p<0.05). The prevalence of ELC had a similar distribution across each aetiopathogenic ischaemic stroke subtype: atherothrombotic (28.6%), cardioembolic (33.3%), lacunar (14.3%) and ESUS (23.8%) (Table 1).” The prevalence of Frank’s sign in patients with an ischemic stroke was 69 / (69+39) = 63.9% and in hemorrhagic 6 / (6+10) = 37.5%, not 92.0% and 8.0% respectively as you mentioned. All calculations must be corrected and then results should be interpreted once again.
- Alcohol intake in binary logistic regression has a value of 0.39 with CI 1.2-1.29 – OR is always included in CI. There is probably a mistype.
- “ELC is associated with a poor outcome (mRS >2) at discharge and at 3 months (76.9% at 3 months). (Table 1 and 2).” – what about of results of adjusted binary logistic regression?
- “Thirty-five patients failed to attend the appointment scheduled for 3 months after stroke” – it would be very interesting what were the reasons of these losses to follow-up. Do you have any additional information?

DISCUSSION

- “The association between ELC and CAD has been reported several times since Sanders T. Frank noticed that many patients with ischaemic heart disease had an earlobe crease [3].” – reference number 3 is just the one article published only 3 years after Frank’s case series. It would be much more informative to potential readers to cite up-to-date reviews.
- “We also found an association of ELC with classical vascular risk factors involved in atherosclerotic disease, especially with hypertension, dyslipidemia and diabetes, as well as with a greater intima-media thickness and presence of carotid plaques [9,22,23].” – as seen in Table 1, there was no association of ELC with dyslipidemia. Did you evaluated intima-media thickness in your patients? There is no information about it in your manuscript. How references number 9, 22 and 23 are related to that sentence?
- “An inverse association with smoking and alcohol intake was found, where non-smokers and patients without alcohol intake presented more ELC. This could be explained by the fact that smokers and patients with alcohol intake of more than 20 gr/day were younger than the global population.” – you performed binary logistic regression adjusted for some variables. In this analysis arterial hypertension, smoking status, alcohol intake, presence of atherosclerotic plaque, and mRS were not significant factors basing on confidence intervals and p-values. I think it is important to mention it after presentation of just simple associations.
- “Although ELC has been traditionally associated to atherotrombosis…” – ELC has been rather associated to atherosclerosis. Atherothrombosis is atherosclerotic lesion damage with thrombus formation linked to acute coronary syndromes or strokes.
- Figure 2 is hard to interpret. Could you describe what is presented on X axis – is it percentage? Is it mean for age? Why there are no other categories – females, no arterial hypertension etc.?
