# Supplementary material for: Prevalence of the Frank’s sign by aetiopathogenic stroke subtype: A prospective analysis
Source: PLoS One. 2021 Dec 15;16(12):e0261080. doi: 10.1371/journal.pone.0261080 (PMC8673637; doi:10.1371/journal.pone.0261080)
Supplement: Attachment — Submitted filename: Response to Reviewer #1 and #2.docx [file pone.0261080.s003.docx]

**ANSWER TO REVIEWER #1**

Dear Prof

We are grateful for your comments and suggestions that will clearly improve the final version of the manuscript.

A point-by-point response to the comments attacheded. We would be pleased to provide additional information if requested and to consider further modification of the text if it should be thought necessary.

ABSTRACT

- “Many studies have associated this sign with heart disease…” - I suggest you to be more precise in abstract and change it to “coronary artery disease”.

o	We have made this change to the abstract as suggested.

Later in the text more specific terms such as "ischaemic heart disease” and "coronary heart disease" are used.

INTRODUCTION

- In the *Introduction* you stated: “The Frank’s sign or ear lobe crease (ELC) is a cutaneous clinical sign (…) covering at least 1/3 of the ear lobe”. I definitely do not agree with that sentence. There is no one clear definition and many authors utilized various ones, including many thresholds of relation of earlobe crease to the whole earlobe. I suggest you to mention this issue in your article.
  - The most widely used definition considers a Frank' sign as a diagonal crease in the ear lobe extending from the tragus across the lobule to the rear edge of the auricle with an angle of 45º in varying depths. The Frank himself describeb the sign as a prominent crease in the lobule portion of the auricle. In clinical practice it is clear and we agree with reviewer 1 that considers the definition of >1/3 of the ear lobe as not being useful and confusing. In fact, it is frequently bilateral affecting all the ear lobule and the variations are mainly in the depth. The identification of ELC particularly in stroke patients who are older than patients with ischaemic heart disease, is very easy. In our series, we have an excellent kappa index inter and intra observer (>0.85). In a few cases we have had doubts about the presence or not of the Frank' sign, usually in unilateral cases where depth is slight and where the whole lobule is affected.
  - We agree that it is interesting to mention this issue in our article, clarify the definition and delete reference to 1/3 of the earlobe.
- “Several studies have considered a possible link between ELC and CAD, and many studies have shown that ELC can serve as a marker of atherosclerotic disease and as a sign of an elevated risk of coronary heart disease in asymptomatic individuals [3-5].” – I suggest you to cite one of the recent reviews on this topic. References 3 to 5 are from 1977, 1974, and 1983, and many new articles have been published since then.
  - We have updated the references, including recent contributions in this field of study. We have not modified the discussion in any relevant way since they do not provide additional information in relation to the objective of our study.
- In the *Introduction* you raised the topic of pathophysiology and development of ELC. I suggest you to read the new, interesting article (Stoyanov, et al. The Histological Basis of Frank's Sign. Head Neck Pathol. 2021;15(2):402-7. doi: 10.1007/s12105-020-01205-4) about it. You may consider to add it to references.

o	We are grateful for the suggestion. We have now quoted from and cited this paper. We have added a paragraph to the introduction section in which the main design and the results of this interesting work are referred to.

- “Some studies have described an association between Frank's sign and the presence of diabetes, hypertension, myocardial infarction and coronary artery disease in patients of both sexes.” – in reference number 4 there is “the prevalence of diabetes mellitus and hypertension was similar in both those with and without the ear crease” and in reference number 9: “Among the different CRFs significant associations were found only between ELC and hypertension (p<0.001) and diabetic retinopathy (p<0.05)”.
  - As stated in the aforementioned paragraph, most studies in this field have described a strong association between the Frank's sign and the presence of classical stroke risk factors such as diabetes and hypertension and particularly with ischaemic heart disease. We agree with the reviewer that the references selected do not provide resounding support to our affirmation. We have now retouched the paragraph to clarify the information being referenced and we have included references that better support these findings.
- “However, only a few studies have focused on the association between the presence of ELC and cerebrovascular disease.” – add references, please.
  - We have now add the appropriate references to justify this sentence.
- “Additionally, carotid artery intima-media thickness (IMT) and atherosclerotic plaques in the carotid arteries have also been described in association with ELC.” – add reference, please.
  - We have now added the appropriate reference here.
- “Therefore, according to these findings, the presence of ELC could serve as a reliable marker of systemic atherosclerosis [11-15].” – reference number 13 (Ntaios, G. Embolic stroke of undeterminated source. Jacc. 2020;75:333-340) seems to be not related.
  - The reviewer's impression is correct. The reference does not correspond to the statement made in this paragraph. We have reviewed and corrected errors in the references.

MATERIAL AND METHODS

- What was the time range when patients had been enrolled? Add this in *Material and methods* section, please.
  - Patients were included over a three-month period 2019 (the 1st patient in the registry was on 16th September 2019).

We have added the inclusion period of patients in the “Material and methods” section.

- What were the “potential confounding factors for the evaluation of ELC…”. Specify this in *Material and methods* section, please.
  - We have included additional information regarding confounding factors that could interfere with the correct evaluation of the Frank's sign. This situation was infrequent, essentially the prolonged use of earrings that had markedly deformed the ear lobe (3 women), some isolated case of ear lobe injury (1 patient), and tattoos (1 patient).
- What do you exactly mean by “principal”, “secondary”, and “tertiary” variables in *Material and methods* section?
  - On reflecting further on this matter, we believe that the differentiation of the variables into primary, secondary and tertiary does not provide useful information. Nor would it be helpful to classify them as either a dependent variable (Frank's sign) or independent variables (hypertension, diabetes mellitus, ...), which would be methodologically sounder from a statistical point of view. We have now deleted this paragraph as we believe that the objective and analysis that is carried out are clear without these distinctions.
- I suggest you to add references to NIHSS, mRS.
  - We have now added appropriate references for the NIHSS and the mRankin Scale.
- Is the protocol of this study available online?
  - The protocol is not available online. This research started out as an end of degree project in the final year of medicine and a first version was made that led on to the current study.

STATISTICAL ANALYSIS

- “Results are expressed as (…) mean ± standard deviation (SD) for continuous variables depending on whether or not they are normally distributed.” – so, how did you presented continuous variables not normally distributed?
  - We have completed the "Statistics" section. Continuous variables are expressed as mean ± standard deviation (SD) and compared with the Student's t test, or median and quartiles and using the Mann-Whitney tests, depending on whether the distribution of the variable was normal (t test) or not (Mann-Whitney). We evaluated the normal distribution of the variables using the Kolmogórov-Smirnov test.
- In *Statistical analysis* section you noted: “The importance of ELC in all stroke subtypes and specifically in ESUS was assessed by binary logistic regression analysis…” and then in Table 1 in *Results* there is “Adjusted mean difference (95% IC)” that is other measure than ORs. Could you explain it to me?
- After bivariate analysis we performed a binary logistic regression model to evaluate the probability of outcome of the Frank's sign (categorical dependent variable) adjusted by all other independent variables that were statistically significant in the bivariate analysis.

To show the results more clearly, we have presented both the bivariate analysis and the multivariate analysis (logistic regression analysis) in the same table (Table 1). The column "adjusted mean difference (95% IC)" gives the classical OR, 95% IC (adjusted by significant independent variables in bivariate analysis).

We have now divided Table 1 into two different tables. Table 1 now shows only the bivariate analysis whereas Table 2 shows the logistic regression analysis (see below). It is to be hoped that this will make it easier for the reader to identify the variables that remain significantly and independently associated with the Frank's sign.

*Table 2. Adjusted odds ratios of ELC for significant clinical variables in bivariate analysis*

|  | **O.R.** | **95% C.I.** | ***p*** |
| --- | --- | --- | --- |
| *Age* | 1.07 | 1.01-1.12 | 0.01 |
| Stroke subtype | 0.51 | 0.04-5.96 | n.s. |
| Arterial hypertension | 2.33 | 0.68-8.10 | n.s. |
| Diabetes mellitus | 5.8 | 1.15-29.49 | <0.05 |
| *Smoker* | 1.3 | 0.34-4.90 | n.s. |
| Alcohol intake | 0.39 | 0.12-1.29 | n.s. |
| Presence of atherosclerotic plaque | 0.71 | 0.17-2.95 | n.s. |
| mRankin ≤ 2 at discharge | 1.24 | 0.29-5.38 | n.s. |
| mRankin ≤ 2 at 3 months | 2.28 | 0.41-12.70 | n.s. |

Age was included as a continuous variable and so the 7% increased association risk is by every 1 year of age increase. Categorical variables were included as 1=yes or 0=no. ELC, or ear lobe crease (Frank’s sign).

RESULTS

- There is one crucial error in calculations presented through the whole manuscript. In *Abstract* you stated: “The Frank’s sign (…) was more prevalent in patients with an ischemic stroke (92.0%). Similar prevalence was found in the different ischaemic stroke subtypes.”. Then, in *Results* section you continued with: “There were no relevant differences in the prevalence of ELC by stroke subtypes.”, “In comparison with ischaemic stroke, we found a low prevalence of the Frank’s sign in haemorrhagic stroke (92.0% vs. 8.0%, p<0.05). The prevalence of ELC had a similar distribution across each aetiopathogenic ischaemic stroke subtype: atherothrombotic (28.6%), cardioembolic (33.3%), lacunar (14.3%) and ESUS (23.8%) (Table 1).” The prevalence of Frank’s sign in patients with an ischemic stroke was 69 / (69+39) = 63.9% and in hemorrhagic 6 / (6+10) = 37.5%, not 92.0% and 8.0% respectively as you mentioned. All calculations must be corrected and then results should be interpreted once again.
  - In fact, there is no calculation error but rather a serious problem with how we have presented the results, which has led to reviewer #1’s understandable confusion. We will try to explain where this mistake lies and why the statistical analysis and interpretation are correct.

As you can be seen in the attached original analysis in SPSS (screenshot) we have expressed the results (ischaemic vs. haemorrhagic using the “% within column” (92.0% vs. 8.0%) rather than “% within row” (63.9% vs. 37.5%).

The results and interpretation are identical (p=0.044), but we can improve how the results are shown in this case.

We have changed the % in Table 1 recording the “% within row” rather than “% within column”, so reflecting more clearly the result and the message as the reviewer detected and stated. The absolute number of patients is identical in each grid and does not change.

We have revised the Abstract and Results section to make the following information clear:

- Prevalence of the Frank’s sign in patients with an ischaemic stroke: 63.9%
- Prevalence of the Frank’s sign in patients with haemorrhagic stroke: 37.5%
- Prevalence of the Frank’s sign in each aetiopathogenic ischaemic stroke subtype: atherothrombotic (69.2%), cardioembolic (60.0%), lacunar (64.3%) and ESUS (65.2%)

The same situation has occurred with the prevalence of the Frank’s sign by aetiopathogenic stroke subtype. See the source document just below.

As suggested by reviewer #1, it is probably clearer to reflect the results using the “% within row” rather than “% within column”. In the case of “% within column” (as reflected in the original submitted version) we can see the % of every stroke subtype in patients with and without the Frank’s sign. Using “% within row” (100% in horizontal), we can see the % of the Frank’ sign in every stroke subtype, which we agree with reviewer #1 is clearer to understand.

We have changed % in Table 1 and the Results section. The absolute number of patients is identical in every grid and does not change. The interpretation does not change.

- Alcohol intake in binary logistic regression has a value of 0.39 with CI 1.2-1.29 – OR is always included in CI. There is probably a mistype.

This slip has now been corrected. It now reads:

**OR 0.39; 95% CI, 0.12 to 1.29; p=n.s.**

Rather than:

OR 0.39; 95% CI, 1.2 to 1.29; p=n.s.

- “ELC is associated with a poor outcome (mRS >2) at discharge and at 3 months (76.9% at 3 months). (Table 1 and 2).” – what about of results of adjusted binary logistic regression?
  - As we can see in Table 2 (multiple logistic regression analysis from Table 1, with the Frank's sign as the dependent variable), the mRankin scale at discharge or at 3 months is not independently associated with the presence of the Frank's sign.
  - We analysed predictors of dependency both at discharge and at 3 months (mRankin >2).

In the paper (Table 3) we included only the bivariate analysis about the mRankin scale at 3 months, although we performed a logistic regression analysis of the mRankin both at discharge and at 3 months, analyzing the variables that independently predict dependency at discharge and at 3 months (mRankin as the dependent variable).

The Frank's sign is not an independent predictor of poor functional outcome (mRankin >2) neither at discharge nor at 3 months.

We attach below the logistic regression analysis from Table 3 in the draft. We have not included its analysis in the original work because we consider that it does not provide relevant information.

As you can see, only age remains as an independent predictor of the Frank’s Sign (8% for every year in age increase, as age is included as a continuous variable).

- We have included a comment in the draft to make it clear that in multivariate analysis, only age remains as an independent predictor of mRankin.

If thex reviewer finds it interesting to include this analysis in the paper or as supplementary material, we can do so.

- “Thirty-five patients failed to attend the appointment scheduled for 3 months after stroke” – it would be very interesting what were the reasons of these losses to follow-up. Do you have any additional information?
  - We have little information about the reasons for not attending the scheduled visit.

We reviewed the computerized medical record used in our health system, which includes all visits either in primary care, in our hospital (other specialist) or in any other hospital in the health network and also includes information in the event of death. In the case of death, we had already recorded the mRankin as 6 in the analysis at 3 months.

- Common reasons in our area are:

- Excellent recovery, which usually demotivates patients from attending the follow-up review (n = 20, mRankin 0-2 [57.1%]).

- In 3 patients, it was due to a change of address to another health area.

- Another common cause is that our health region covers a very large geographical area and patients/relatives sometimes perform the follow-up visit with neurologists from nearby hospitals, especially in patients with greater sequelae (n = 12)

We did not attempt to contact the patients who did not attend the 3-month visit personally as we believe that the results would not be different with respect to the relationship between the Frank’s sign and Rankin >2:

- The Frank's sign is not an independent predictor of poor functional outcome (mRankin> 2) neither at discharge (the whole population) nor at 3 months.

- We performed the 3-month analysis both with the available sample that attended the visit and with the entire sample of patients, assuming the worst scenario and assigning the mRankin at discharge at the 3-month visit, also without significant changes to the results.

- Finally, we analyzed the clinical profile of the 35 patients not evaluated in person at 3 months (vascular risk factors, prevalence of Frank's sign, stroke subtype, etc.), showing no significant differences with respect to the population that attended the control visit.

DISCUSSION

- “The association between ELC and CAD has been reported several times since Sanders T. Frank noticed that many patients with ischaemic heart disease had an earlobe crease [3].” – reference number 3 is just the one article published only 3 years after Frank’s case series. It would be much more informative to potential readers to cite up-to-date reviews.
  - We have now updated the references.
- “We also found an association of ELC with classical vascular risk factors involved in atherosclerotic disease, especially with hypertension, dyslipidemia and diabetes, as well as with a greater intima-media thickness and presence of carotid plaques [9,22,23].” – as seen in Table 1, there was no association of ELC with dyslipidemia. Did you evaluated intima-media thickness in your patients? There is no information about it in your manuscript. How references number 9, 22 and 23 are related to that sentence?
  - Unfortunately, this is an uncorrected error from an earlier version of the work.

We have corrected the paragraph to reflect the results that we mention and that we correctly set out in the "Results" and "Table 1" sections.

The evaluation of the IMT was included in the 1st draft of our work but eliminated in the final version submitted to PLOS ONE, as the IMT had not been recorded in approximately 50% of patients and was considered unreliable.

The Stroke Unit includes a neurosonology department in which we carried out a systematic ultrasonographic study of admitted patients (supraaortic trunk as well as transcranial colour coded sonography) by an experienced neurologist accredited by the Spanish Society of Neurosonology. The study includes the evaluation of the IMT, but frequently no quantified measurement was collected in the report or it was collected in cases with evident pathology. We do not have a photographic record to reevaluate them.

Despite being very interesting, we decided to eliminate this parameter given that it was not a reliable sample and could be biased.

- “An inverse association with smoking and alcohol intake was found, where non-smokers and patients without alcohol intake presented more ELC. This could be explained by the fact that smokers and patients with alcohol intake of more than 20 gr/day were younger than the global population.” – you performed binary logistic regression adjusted for some variables. In this analysis arterial hypertension, smoking status, alcohol intake, presence of atherosclerotic plaque, and mRS were not significant factors basing on confidence intervals and p-values. I think it is important to mention it after presentation of just simple associations.
  - We have now included a comment in the Results and Discussion sections to clarify and reinforce our results. Additionally, as commented previously, we have divided Table 1 into two different tables. Table 1 now exclusively shows the bivariate analysis whereas Table 2 shows the logistic regression analysis. This will probably highlight more clearly to the reader those variables that remain significantly and independently associated with the Frank's sign.
- “Although ELC has been traditionally associated to atherotrombosis…” – ELC has been rather associated to atherosclerosis. Atherothrombosis is atherosclerotic lesion damage with thrombus formation linked to acute coronary syndromes or strokes.
  - The comment is adequate. In the context of our study and the published literature, the relevance of ELC is mainly as a marker of atheromatosis and not of atherothrombosis. We have changed “atherothrombosis'' to “atherosclerosis”.
- Figure 2 is hard to interpret. Could you describe what is presented on X axis – is it percentage? Is it mean for age? Why there are no other categories – females, no arterial hypertension etc.?
  - The number of patients with cryptogenic stroke is small and the analysis has insufficient statistical power (an aspect that we already highlighted in the text and mentioned as a limitation of the study). It seemed clearer to us to express the results in the form of a graph for the study message (that the Frank's sign in patients with cryptogenic stroke may be helpful in suspecting the underlying cardioembolic or atherothrombotic origin and in guiding the aetiological study in this subgroup of patients) .

We have added further detail to the figure to facilitate its interpretation.

If the reviewers thinks that it is preferable to remove Figure 2, please let us know.

Thanks,

**ANSWER TO REVIEWER #2**

Dear Prof

We are grateful for your comments and suggestions that are a great help in improving the final version of the manuscript.

A point-by-point response to the comments is attached. We would be pleased to provide additional information if requested and to consider further modification of the text if it should be thought necessary.

1.- It should be important to mention in the discussion whether the presence of the unilateral or bilateral Frank's Sign has the same clinical translation according to previous reports, since in the current study both were taken without distinction.

- We have included a comment in the "Discussion" section.

We found no relevant differences in the clinical profile of patients between patients with a unilateral Frank’s sign (25% of patients) and patients with bilateral signs (75% of patients). For this reason, and so as not to lose statistical power, we analysed as Frank’s sign yes/no.

- Below we attach the bivariate analysis comparing the profile of patients with unilateral or bilateral Frank's sign.

In addition to assessing the presence of a unilateral/bilateral Frank's sign, we analyzed some additional variables, which also failed to provide additional information on the consideration of Frank's sign as present/absent. We evaluated the length of the crease (complete/incomplete) and the Grade of ELC (Mild, when it appears as a superficial wrinkling on the earlobe; Moderate when it is a sulcus with a visible base; Severe when the sulcus is so deep that the base is not visible).

A higher degree of the Frank’s sign (greater depth), correlated with older patients and a greater prevalence of vascular risk factors, but without relevant additional finding with respect to the evaluation as presence/absence of the Frank’s sign (there were a collinearity between the Frank’s sign and aspects such as degree, as expected).

|  | **FRANK’s SIGN** | | |  |
| --- | --- | --- | --- | --- |
|  | **UNILATERAL** | **BILATERAL** | **TOTAL** | **p value** |
| Male  Female | 80%  20% | 71.1%  28.9% | 73.3%  26.7% | n.s. |
| Age  < 50  51-59  60-69  70-79  >80 | 6,7%  6,7%  0%  53.3%  33.3% | 8.9%  4.4%  13.3%  40%  33.3% | 8.3%  5.0%  10.0%  43.3%  33.3% | n.s |
| Arterial hypertension | 73.3% | 84.4% | 81.7% | n.s |
| Diabetes Mellitus | 40.0% | 37.8% | 38.3% | n.s |
| Dyslipidemia | 46.7% | 57.8% | 55.0% | n.s. |
| Obesity (BMI>30) | 28.6% | 36.4% | 34.5% | n.s. |
| Smoker  Ex-smoker  Non-smoker | 13.3%  26.7%  60% | 24.4%  28.9%  46.7% | 21.7%  28.3%  50% | n.s |
| Alcohol intake ( >20gr/d ) | 6.7% | 15.6% | 13.3% | n.s |
| Ischaemic heart disease | 40% | 31.1% | 33.3% | n.s |
| Previous ischaemic stroke | 14.3% | 18.6% | 17.5% | n.s. |
| Presence of non-stenotic atherosclerotic plaque | 53.8% | 63.2% | 60.8% | n.s |
| Internal carotid artery stenosis (>50%) | 35.7% | 25.6% | 28.3% | n.s. |
| Stenosis other arteries | 21.4% | 28.2% | 26.4% | n.s. |
| mRankin ≤ 2 prior to index stroke | 100% | 91.1% | 93.3% | n.s |
| mRankin ≤ 2 at discharge | 73.3% | 46.7% | 53.3% | n.s |
| mRankin ≤ 2 at 3 months | 66.7% | 61.8% | 62.8% | n.s |

2.- In the paragraph: As previously described, we found a strong and significant association between the presence of ELC and age, with ELC being particularly prevalent in patients who were older than 70 years (74.7%). Does the term “as previously” mean in previous studies? Because it is the first time that this idea was mentioned in the results of the body of the text.

- This paragraph refers to previously published literature, in which a close association is systematically found between the Frank's sign and increased age, as in our work. We have now improved the wording and included the appropriate references to make the sense absolutely clear.

3.- In the paragraph: On analysing carotid ultrasonographic markers of vascular atherosclerosis, we found a significant relationship between the prevalence of ELC and the presence of atherosclerotic plaques (63.6% vs. 45.2%, p<0.05). Are you referring to non-stenotic plaques? It should be important to clarify.

- Yes, here we are referring to non-stenotic atherosclerotic plaques. We have now improved the sentence to make this clear. Table 1 shows the results for atherosclerotic plaques, where we have now also added that they are non-stenotic, and carotid stenosis.

4.- In the limitations of the study, it should be important to included that the assessment of Frank's sign was carried out through photographs.

- In fact, it was evaluated by a vascular neurologist during the visit to the Stroke Unit, usually on the day of admission. In addition, after the consent of the patient or family was obtained, photographs of both ears were taken in order to be able to carry out an additional evaluation by a neurologist blinded to the clinical condition and the aetiology of the patient or to evaluate the inter-intra-observer variability. They are still archived and available.

We have now added a sentence in this respect in the limitations section, although we chose this methodology consciously as a potential strength of the study (mainly to maintain blind evaluation of ELC with regards to the aetiopathogenic cause of stroke).

5.- It should be important to improve the quality of the tables, especially Table 2, which has disordered information ("Internal carotid artery stenosis")

- We have now worked on improving the appearance and presentation of the information in the tables.

6.- I think that the conclusion could be improved if the relevance of investigating atherosclerosis in patients with ESUS and Frank's sign is pointed out.

- We agree with the reviewer's comment. We have now reinforced the message of our work.

Thanks,
